# Antimicrobial Resistance and Environmental Health

**DOI:** 10.3390/antibiotics14020113

**Published:** 2025-01-21

**Authors:** Akebe Luther King Abia

**Affiliations:** 1 Antimicrobial Research Unit, College of Health Sciences, University of KwaZulu-Natal, Durban 4000, South Africa; lutherkinga@yahoo.fr; 2 Environmental Research Foundation, Westville 3630, South Africa

If left unaddressed, the casualties due to or associated with antimicrobial resistance (AMR) may surpass those associated with all pandemics humanity has ever experienced. Hence, the recent United Nations General Assembly (UNGA) High-Level Meeting on AMR saw all attending heads of state recognising, through a political declaration, that AMR was a global threat requiring immediate and sustainable solutions [1]. An important aspect of this declaration is its reiteration of the need for a One Health approach to addressing the AMR crises, with the environment being recognised more than ever as a significant contributor [1]. Although the first high-level meeting occurred in 2016, almost a decade ago [2], most studies have focused on the human and animal sectors with little attention to the environment. However, studies have shown that the environment is an important reservoir and potential source of AMR [3,4]. This Special Issue (SI) presents manuscripts covering different aspects of the environmental dimension of AMR, ranging from the AMR on inanimate household objects and vegetables in the field to the impact of anthropogenic activities on the presence of antibiotics in the environment and its associated effects on AMR development and evolution. It also presents research on genomic approaches to elucidate AMR in environmental bacteria, the role of wastewater as a proxy to understand AMR in communities, bioaerosols as vehicles for the spread of AMR is considered and a community-centric approach to addressing AMR. Eleven articles by 102 authors from Africa, Europe, America and Asia were finally published after rigorous peer review.

Antibiotic discovery changed the face of modern medicine. However, this victory over human infections was short-lived as microorganisms became resistant through diverse mechanisms and evolved to include multidrug-resistant species identified using different methods from culture to genomics, as reviewed by Salvarjan et al. The authors also revealed the ubiquity of these organisms in various environments and mentioned their ecological and human health implications and ways to address AMR.

Although AMR is a natural process, it is well recognised that its occurrence is amplified due to the excessive use of pharmaceuticals in human and animal health. This results in the massive discharge of these chemicals in the environment in partially or unmetabolised forms that are still active and cause selective pressure, resulting in the development of AMR in environmental bacteria, as indicated by Salvarjan. This is further reiterated by Amangelsin et al., who reviewed the impact of tetracycline pollution on aquatic environments and the strategies used to remediate such environments. The authors observed that tetracycline could accumulate in the food chain, causing adverse effects such as intoxication of environmental microorganisms, promoting the development and dissemination of AMR, threatening drinking and irrigation water quality, and disrupting the human microbial flora upon consumption. However, tetracycline can be degraded into environmentally friendly components through physical, chemical, and biological processes.

Farkas et al. also showed that anthropogenic activities led to the spread of antibiotic-resistant enterococci in aquatic milieus (wastewaters, hospital effluents, surface waters and groundwater). Furthermore, the isolates carried several antibiotic resistance genes and displayed a high heterogeneity. Similarly, Irfan et al. showed that hospital and municipal wastewater contained clinically significant Gram-negative bacteria harbouring extended-spectrum β-lactam and carbapenem resistance genes.

Apart from the dissemination of resistant species, the discharge from poorly treated or untreated wastewater from hospitals or municipalities leads to the pollution of rivers. This can further lead to new AMR threats, as demonstrated by Fono-Tamo et al., who investigated resistance in *Aeromonas rivipollensis*, an emerging pathogen associated with numerous human infections. The authors used whole genome sequencing to reveal the presence of beta-lactams, cephalosporin, and tetracycline resistance genes. The presence of several mobile genetic elements indicates that these organisms can easily transfer these resistance traits to other organisms in the environment. For example, this can happen when polluted water is used to irrigate farms. This was demonstrated by Riva et al. The authors demonstrated that an environmental *Acinetobacter baylyi* BD413 strain could acquire extracellular DNA harbouring resistance genes on lettuce. Although they used a simulation, the authors showed that experimental conditions were similar to real-life scenarios.

Most informal settings, especially in low- and middle-income countries, rely on polluted rivers and streams for daily water needs. Without access to portable water and waste disposal systems, hygiene conditions are usually poor, resulting in the transmission of microorganisms, including drug-resistant ones, between people and their surroundings. Rakhalaru et al. showed that household fomites like kitchen cloths and toilet surfaces harboured antibiotic-resistant pathogenic *E. coli*, which could be transmitted to users of these materials and surfaces, calling for improved hygiene practices within communities to safeguard public health.

Sewage discharge into the aquatic environment also has implications for the marine environment. Al-Sawari et al. used genomic methods to characterise resistance traits in *E. coli* from molluscs and coastal water. They found genes conferring resistance to multiple antibiotic classes, and a correlation existed between these genes and the phenotypic resistance observed. The presence of plasmids in the isolates calls for monitoring gene transfer in the aquatic environment.

Although the environmental dimension of AMR has gained considerable attention recently, other matrices like air are not usually the research focus. This creates gaps in the quest to understand the complete environmental resistome. With limited research in this field, George et al. revealed that many human activities, including wastewater treatment and agriculture, generate bioaerosols that can be airborne over long distances. The authors presented ongoing projects to elucidate AMR in this understudied environmental compartment.

Conducting epidemiological studies to map the resistome within communities is challenging. Thus, wastewater-based epidemiology has emerged as an approach to characterise the resistome of communities by analysing AMR in wastewater. However, while this approach is feasible in most developed countries, this is not the case with low- and middle-income countries where many challenges persist, like the lack of sewage networks, appropriate trained personnel, and funding to conduct such studies. Although such studies are conducted globally, several gaps regarding methodologies and reporting approaches still exist, calling for standardised protocols for effective wastewater-based AMR research. This was the focus of the review presented by Abia et al.

The One Health concept has been highly advocated for addressing AMR globally. However, measuring ABR interventions’ performance in communities is challenging due to the lack of objective criteria. Using acquired community engagement experiences, Mathew et al. proposed and piloted an indicator framework of 15 points based on prioritisation. Their study found that implementing AMR interventions yielded varying results based on the community concerned and suggested that the community-centric approach should be incorporated into AMR National Action Plans to better understand the global AMR landscape vis à vis interventions.

Overall, this Special Issue of Antibiotics presents crucial aspects of AMR, ranging from its origin, evolution, dissemination, consequences, and mitigation strategies. With the recent political declaration on AMR, it is hoped that the contributions in this Special Issue will stimulate more in-depth discussion and research on the environmental dimension of AMR and its implications on human and animal health.

## List of Contributions

1. Mathew, P.; Chandy, S.J.; Sivaraman, S.; Ranjalkar, J.; Ali, H.M.; Thomas, S.A. Formulating a Community-Centric Indicator Framework to Quantify One Health Drivers of Antibiotic Resistance: A Preliminary Step towards Fostering ‘Antibiotic-Smart Communities’. *Antibiotics* **2024**, *13*, 63. https://doi.org/10.3390/antibiotics13010063.
2. Al-Sarawi, H.A.; Habibi, N.; Uddin, S.; Jha, A.N.; Al-Sarawi, M.A.; Lyons, B.P. Antibiotic Resistance Mediated by *Escherichia coli* in Kuwait Marine Environment as Revealed through Genomic Analysis. *Antibiotics* **2023**, *12*, 1366. https://doi.org/10.3390/antibiotics12091366.
3. Rakhalaru, P.; Munzhedzi, L.; Abia, A.L.K.; Kabue, J.P.; Potgieter, N.; Traore, A.N. Prevalence and Antimicrobial Resistance Profile of Diarrheagenic *Escherichia coli* from Fomites in Rural Households in South Africa. *Antibiotics* **2023**, *12*, 1345. https://doi.org/10.3390/antibiotics12081345.
4. Irfan, M.; Almotiri, A.; AlZeyadi, Z.A. Antimicrobial Resistance and β-Lactamase Production in Clinically Significant Gram-Negative Bacteria Isolated from Hospital and Municipal Wastewater. *Antibiotics* **2023**, *12*, 653. https://doi.org/10.3390/antibiotics12040653.
5. Fono-Tamo, E.U.K.; Kamika, I.; Dewar, J.B.; Lekota, K.E. Comparative Genomics Revealed a Potential Threat of *Aeromonas rivipollensis* G87 Strain and Its Antibiotic Resistance. *Antibiotics* **2023**, *12*, 131. https://doi.org/10.3390/antibiotics12010131.
6. Riva, V.; Patania, G.; Riva, F.; Vergani, L.; Crotti, E.; Mapelli, F. *Acinetobacter baylyi* Strain BD413 Can Acquire an Antibiotic Resistance Gene by Natural Transformation on Lettuce Phylloplane and Enter the Endosphere. *Antibiotics* **2022**, *11*, 1231. https://doi.org/10.3390/antibiotics11091231.
7. Farkas, A.; Coman, C.; Szekeres, E.; Teban-Man, A.; Carpa, R.; Butiuc-Keul, A. Molecular Typing Reveals Environmental Dispersion of Antibiotic-Resistant Enterococci under Anthropogenic Pressure. *Antibiotics* **2022**, *11*, 1213. https://doi.org/10.3390/antibiotics11091213.
8. Abia, A.L.K.; Baloyi, T.; Traore, A.N.; Potgieter, N. The African Wastewater Resistome: Identifying Knowledge Gaps to Inform Future Research Directions. *Antibiotics* **2023**, *12*, 805. https://doi.org/10.3390/antibiotics12050805.
9. Amangelsin, Y.; Semenova, Y.; Dadar, M.; Aljofan, M.; Bjørklund, G. The Impact of Tetracycline Pollution on the Aquatic Environment and Removal Strategies. *Antibiotics* **2023**, *12*, 440. https://doi.org/10.3390/antibiotics12030440.
10. Selvarajan, R.; Obize, C.; Sibanda, T.; Abia, A.L.K.; Long, H. Evolution and Emergence of Antibiotic Resistance in Given Ecosystems: Possible Strategies for Addressing the Challenge of Antibiotic Resistance. *Antibiotics* **2023**, *12*, 28. https://doi.org/10.3390/antibiotics12010028.
11. George, P.B.L.; Rossi, F.; St-Germain, M.-W.; Amato, P.; Badard, T.; Bergeron, M.G.; Boissinot, M.; Charette, S.J.; Coleman, B.L.; Corbeil, J.; et al. Antimicrobial Resistance in the Environment: Towards Elucidating the Roles of Bioaerosols in Transmission and Detection of Antibacterial Resistance Genes. *Antibiotics* **2022**, *11*, 974. https://doi.org/10.3390/antibiotics11070974.
